# Gold Nanocolumnar Templates for Effective Chemical Sensing by Surface-Enhanced Raman Scattering

**DOI:** 10.3390/nano12234157

**Published:** 2022-11-24

**Authors:** Grégory Barbillon, Christophe Humbert, María Ujué González, José Miguel García-Martín

**Affiliations:** 1 EPF-Ecole d’Ingénieurs, 55 Avenue du Président Wilson, 94230 Cachan, France; 2 Institut de Chimie Physique, Université Paris-Saclay, CNRS, UMR8000, 91405 Orsay, France; 3 Instituto de Micro y Nanotecnología, IMN-CNM, CSIC (CEI UAM+CSIC), Isaac Newton 8, Tres Cantos, 28760 Madrid, Spain

**Keywords:** SERS, sensing, gold, plasmonics, adsorption, thiophenol, GLAD

## Abstract

Herein, we investigate the chemical sensing by surface-enhanced Raman scattering regarding two templates of gold nanocolumns (vertical and tilted) manufactured by glancing angle deposition with magnetron sputtering. We selected this fabrication technique due to its advantages in terms of low-cost production and ease of implementation. These gold nanocolumnar structures allow producing a high density of strongly confined electric field spots within the nanogaps between the neighboring nanocolumns. Thiophenol molecules were used as model analytes since they have the principal property to adsorb well on gold surfaces. Regarding chemical sensing, the vertical (tilted) nanocolumnar templates showed a detection threshold limit of 10 nM (20 nM), an enhancement factor of 9.8 × 10${}^{8}$ (4.8 × 10${}^{8}$), and a high quality of adsorption with an adsorption constant $K_{ads}$ of 2.0 × 10${}^{6}$ M${}^{-1}$ (1.8 × 10${}^{6}$ M${}^{-1}$) for thiophenol molecules.

## 1. Introduction

Surface-enhanced Raman scattering (SERS) is a mighty and responsive spectroscopic instrument with regard to the identification and sensing of chemical and biological analytes [1,2,3,4,5,6,7]. Nanostructured metallic surfaces exhibiting plasmonic properties can enhance the Raman signal thanks to strong confined electric fields (hotspots) generated by the plasmons resonating generally close to the excitation wavelength used for Raman measurements. These confined electric fields may be tailored by tuning the size, shape, and composition of metallic nanosystems [8,9,10,11,12]. A great number of fabrication methods exist to develop plasmonic nanostructures and thus tune these hotspots, such as lithographic techniques [13,14,15,16,17,18,19,20], self-assembly processes [21,22,23,24], and technologies of physical vapor deposition (PVD) [25,26,27,28,29]. However, some lithographic technologies are expensive and their manufacturing time is long. Moreover, in the self-assembly method, several complex steps of chemical processes are implied. Regarding PVD methods, oblique angle deposition (OAD) is a smart strategy for obtaining purely metallic or alloyed nanostructured surfaces that can be employed as SERS substrates [30]. Among PVD methods, magnetron sputtering is the most interesting for industrial applications due to its low-cost and its ease of implementation, thus allowing scaled-up manufacturing on large surfaces [31]. Various metallic nanosystems can be obtained with OAD deposition such as porous nanostructured films [32,33,34,35]. In particular, nanocolumnar films are obtained if the tilt angle (angle between the atomic flux and the normal to the substrate) is above 70${}^{\circ }$, and in this case, the expression glancing angle deposition (GLAD) is commonly used. Such nanocolumnar films are of special interest for SERS due to the confinement and enhancement of the electric field within the nanogaps between neighboring nanocolumns [36]. Various works also used the GLAD or OAD techniques sometimes coupled to other techniques for the fabrication of SERS templates with a plethora of nanosystem geometries such as tilted silver nanorods, armrest Ag nanorods, gold trimers and dimers, metal–insulator–metal nanoparticles, and Ag@Au nanorods, achieving enhancement factors (EFs) of 8 × 10${}^{5}$ to 10${}^{8}$ [36,37,38,39,40,41,42,43,44]. In addition, a large number of research groups have already investigated thiophenol detection by SERS, reaching EFs of 10${}^{2}$ to 10${}^{6}$, and using other manufacturing technologies of plasmonic nanosystems [45,46,47]. Nonetheless, these research groups did not consider in their works the constant of adsorption for obtaining a SERS spectrum. This adsorption constant $K_{Ads}$ is a key parameter corresponding to the efficiency of analyte adsorption on the metallic nanostructured surfaces. $K_{Ads}$ can be determined and extracted from a Langmuir model [48,49,50,51,52].

The goal of this work was to address the efficient detection scheme of thiophenol molecules by SERS through two designs of gold nanocolumnar structures (vertical and tilted) manufactured by GLAD magnetron sputtering (low-cost technique). These templates provide: (i) a high density of strongly confined electric fields within the nanogaps between the neighboring nanocolumns on large surfaces; (ii) large enhancement factors of the SERS signal superior to those obtained in the scientific literature with low-cost manufacturing methods on large areas; and (iii) a high adsorption efficiency of thiophenol molecules on these gold nanocolumnar structures.

## 2. Experimental Methods

### 2.1. Fabrication of the Gold Vertical and Tilted Nanocolumnar Templates

The nanocolumnar films have been fabricated by GLAD with magnetron sputtering in a UHV chamber (base pressure in the 10${}^{-10}$ mbar range) using a magnetron source supplied by AJA (North Scituate, MA, USA) with a circular gold target of 3.8 cm diameter. The geometrical configuration and the deposition parameters were chosen to guarantee that the deposition occurs in the “low-pressure, long-throw” regime that maximizes the ratio of ballistic/thermalized atoms, therefore improving the definition of the nanocolumnar shape [53,54]. The substrate–target distance was 19 cm and a 4.5 cm long cylindrical metallic chimney was put on the target top to increase the collimation of the sputtered atomic flux and trap a huge amount of the thermalized atoms [55]. The sputter gas was Argon. The pressure during the deposition was 1.5 × 10${}^{-3}$ mbar, the lowest value permitting the formation of stable plasma, therefore minimizing the collisions between sputtered atoms and Ar${}^{+}$ ions that may lead to thermalization [56]. Direct current excitation was used at a constant power of 100 W, a value that produced a visible plasma glow that remained more than 10 cm away from the substrate, which is an indication that the deposition was performed in the so-called weak plasma regime [57]. The substrate was initially placed in front of the target (i.e., parallel configuration) and then slanted at an angle of 84${}^{\circ }$ to reach the GLAD condition before deposition was initiated. Glass substrates with 0.75 mm thickness and 8 × 8 mm${}^{2}$ square shape from Präzisions Glas & Optik GmbH (Iserlohn, Germany) were cleaned with acetone, isopropanol, and deionized water, then used. For the sake of obtaining cross-section images, silicon substrates were also used, which were subsequently cut by cleavage. Films with tilted or vertical nanocolumns were fabricated in both cases with the same aforementioned conditions and a deposition time of 20 min. The only additional parameter to achieve vertical nanocolumns was substrate rotation at an angular speed of 3 rpm [58]. Finally, it has to be mentioned that all of the samples have an adhesion layer of Ti (5 nm thick) deposited by magnetron sputtering employing the standard parallel configuration (i.e., without tilt angle between substrate and target) with 100 W during 192 s.

### 2.2. Thiophenol Grafting on Au Vertical and Tilted Nanocolumnar Templates

For measuring the detection threshold limit of Au vertical and tilted nanocolumnar templates, we used thiophenol molecules as a marker because of their adsorption quality on the gold surface [59,60]. In the first place, thiophenol solutions (in ethanol) with concentrations from 10 nM to 1 mM were prepared. Afterwards, Au nanocolumnar templates were plunged in the solution for a period of 24 h accompanied by rinsing with pure ethanol and subsequent drying with nitrogen. For experiments on a glass substrate without any gold nanocolumns (serving as reference), a 1 M concentration of thiophenol was employed.

### 2.3. Extinction and Raman Spectroscopies

To record extinction spectra, a spectrophotometer from Agilent (Cary-5000) configured in normal transmission was used. The extinction (E) spectrum was obtained by taking the following formula E(λ) = log${}_{10}$ ($I_{0}/I$), where $I_{0}$ and *I* are the transmitted intensities through the glass substrate (reference) and the gold nanocolumnar templates, respectively. Regarding the SERS measurements, we used a spectrophotometer (Labram) from Horiba Scientific (Kyoto, Japan) whose the spectral resolution is 1 cm${}^{-1}$, and two excitation wavelengths ($\lambda_{exc}$) at 532 nm (power = 2.3 mW) and 633 nm (power = 1.1 mW). The acquisition times have been set at 5 s and 10 s, respectively. We concentrated each excitation laser on the gold nanocolumnar templates via a microscope objective (N.A. = 0.9; ×100). This latter also allowed the collection of SERS signals from these templates. For experiments on glass substrate without any gold nanocolumns (serving as reference), the same parameters of excitation were implemented. All the SERS and Raman spectra were divided by the laser power and the acquisition time for comparative uses.

## 3. Results and Discussion

**Figure 1:**
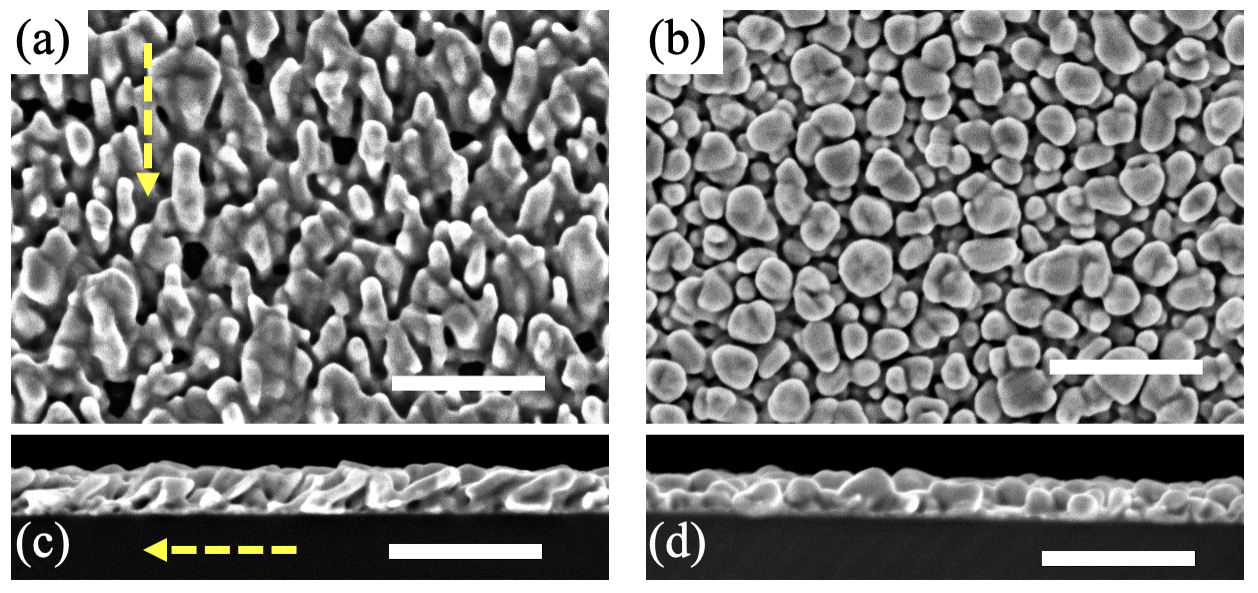
SEM characterization of the fabricated nanocolumnar templates: top-view images with (**a**) tilted and (**b**) vertical nanocolumns, and cross-section micrographs with (**c**) tilted and (**d**) vertical nanocolumns. For all SEM images, the white scale line is 250 nm. The yellow-dotted arrow in (**a**,**c**) indicates the direction of the atomic flux during gold deposition.

Regarding our sensing study, we compared two types of gold nanocolumnar templates: (i) with tilted and (ii) with vertical nanocolumns. The templates were fabricated using GLAD with magnetron sputtering, as described in Section 2.1, adding substrate rotation in the latter case. Figure 1 shows the representative SEM images of both templates. Besides the slanted or vertical orientation of the nanopillars that are randomly placed onto the glass substrate, another important difference can be observed. The atomic shadowing effect that is responsible for the development of nanocolumns in the GLAD configuration only takes place in the direction of the incoming atomic flux. As a result, when the substrate is fixed, coalescence may appear in the perpendicular direction to such flux when the nanocolumns grow. This is the case of the template with tilted nanocolumns, as shown in Figure 1a, where some nanocolumns coalesce in the direction perpendicular to their axis, the axis being defined by the atomic flux direction that is marked by the yellow dotted arrow. In clear contrast, when the substrate rotates during the deposition, the shadowing effect is isotropic and the coalescence is reduced, giving rise to much better defined individual nanostructures with vertical orientation (see Figure 1b).

### 3.1. Plasmonic Properties of the Gold Vertical and Tilted Nanocolumnar Templates

**Figure 2:**
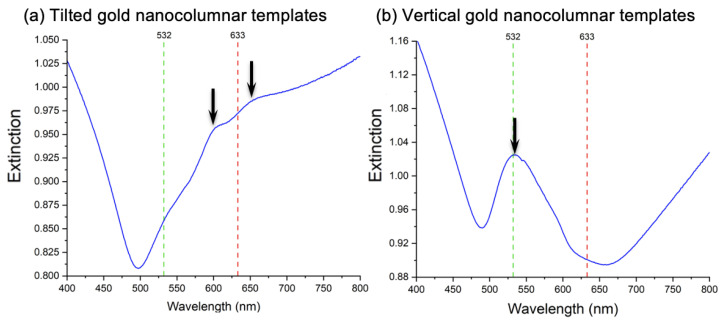
Extinction spectroscopy measurements for (**a**) tilted and (**b**) vertical gold nanocolumnar templates. The green- and red-dashed lines coincide with $\lambda_{exc}$ of 532 nm and 633 nm, respectively, used in the subsequent SERS experiments. The black arrows mark the resonance positions.

To find the plasmon resonances of Au vertical and tilted nanocolumnar templates, we recorded their extinction spectra (see Figure 2). For the tilted gold nanocolumns, we found two plasmonic resonances at 605 nm and 655 nm. The second plasmon resonance is relatively near to $\lambda_{exc}$ of 633 nm (Figure 2a). For the vertical gold ones, we found a plasmonic resonance around 534 nm close to $\lambda_{exc}$ of 532 nm (see Figure 2b). Moreover, the shape of the extinction curves for the tilted and vertical nanocolumnar templates are clearly different, and this fact can be related to their respective morphologies. On the one hand, the clear peak at 534 nm exhibited in Figure 2b by the template with vertical nanocolumns can be ascribed to the excitation of a well-defined localized surface plasmon of individual nanostructures. On the other hand, the optical response of tilted nanocolumns shows the same trend as a continuous gold film [58] but with two small peaks superimposed (Figure 2a). It can be said that this tilted nanocolumnar template, due to the strong coalescence of neighboring columns, behaves as a nanocorrugated gold film, and those small peaks can be associated with plasmonic resonances of the nanoscale roughness.

### 3.2. SERS Performance of the Gold Vertical and Tilted Nanocolumnar Templates

**Figure 3:**
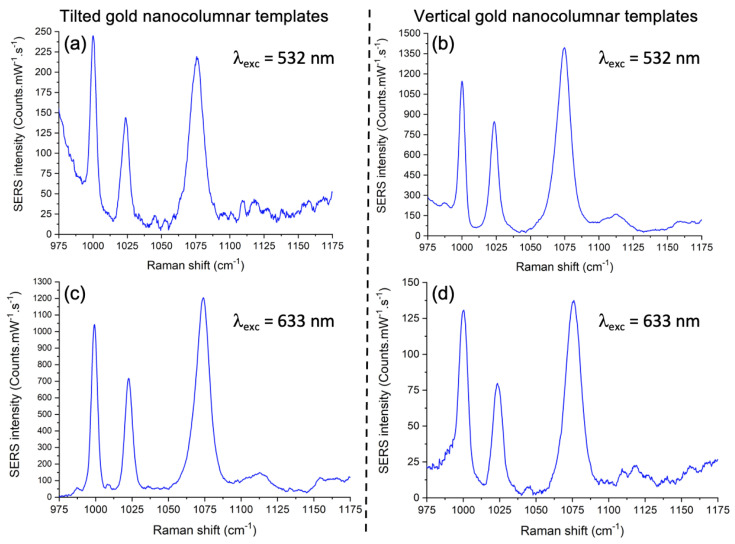
SERS spectra of thiophenol (1 mM) obtained with the tilted (**left panels**) and vertical (**right panels**) gold nanocolumnar templates for the two excitation wavelengths: (**a**,**b**) at 532 nm and (**c**,**d**) at 633 nm.

To measure the detection capability of Au vertical and tilted nanocolumnar templates, we functionalized them with thiophenol by using the protocol depicted in Section 2.2. Then, we recorded SERS spectra at two excitation wavelengths, 532 nm and 633 nm. From the SERS spectra at the concentration of 1 mM displayed in Figure 3, three Raman peaks are well distinguished, which are characteristic of thiophenol molecules [61,62] (see Table 1).

For the SERS template composed of tilted Au nanocolumns (see Figure 3a,c), we observed 5 times higher SERS intensities for the three Raman peaks recorded with the $\lambda_{exc}$ of 633 nm than for those excited at 532 nm due to the fact that one plasmon resonance is very close to the $\lambda_{exc}$ of 633 nm. On the contrary, for vertical Au nanocolumns (see Figure 3b,d), the SERS intensities obtained with the excitation wavelength of 532 nm are 10-fold higher compared to those at 633 nm owing to the fact the plasmon resonance is close to the $\lambda_{exc}$ of 532 nm. It is worth mentioning that, in a previous work combining scanning near-field optical microscopy with finite-difference time–domain simulations, it has been shown that the enhanced response of these nanocolumnar templates was due to the existence of hotspots that are localized in the gaps between neighboring nanocolumns [36]. Considering that the SERS signal increases in each kind of template when the excitation wavelength is closer to its plasmonic resonance, we can deduce that these hotspots are associated with the resonances, with their intensity being stronger when we are closer to them.

**Figure 4:**
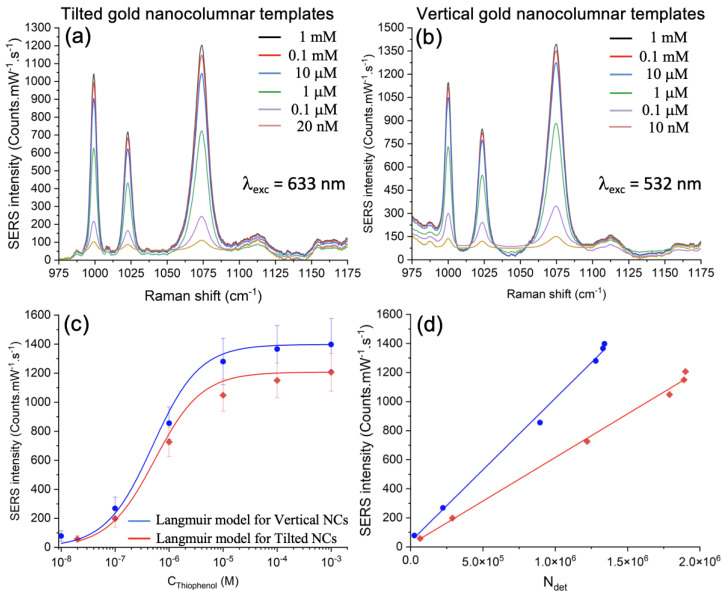
SERS spectra of thiophenol on the gold nanocolumnar templates with (**a**) tilted and (**b**) vertical nanocolumns for concentrations ranging from 10 nM to 1 mM. (**c**) Intensity of SERS signal versus thiophenol concentration ($C_{Thiophenol}$) for vertical (blue points) and tilted (red diamonds) nanocolumns. The blue and red curves represent the Langmuir fits for vertical ($R^{2}=0.984$) and tilted ($R^{2}=0.995$) nanocolumns, respectively. (**d**) Intensity of SERS signal versus the number of detected molecules $N_{det}$ (thiophenol) for vertical (blue points) and tilted (red diamonds) gold nanocolumnar templates. The blue and red lines correspond to the linear fits whose linear equations are y=0.00098x+37.95 ($R^{2}=0.995$) and y=0.00059x+15.69 ($R^{2}=0.996$), respectively.

In order to fix the detection threshold limit of thiophenol molecules, we chose the most intense Raman peak (1073 cm${}^{-1}$), the excitation wavelength of 532 nm for vertical gold nanocolumns and 633 nm for tilted gold nanocolumns. We measured SERS spectra for thiophenol concentrations ranging from 10 nM to 1 mM (see Figure 4a,b). Then, we displayed the SERS intensity at the selected peak in each case versus the thiophenol concentration (see Figure 4c, wherein blue and red colors correspond to vertical and tilted gold nanocolumns, respectively). Detection threshold limits (LODs) of 10 nM and 20 nM were reached for vertical and tilted gold nanocolumns, respectively. Moreover, we evaluated the uniformity of the SERS signal for Au nanocolumnar templates by acquiring the intensity of the SERS signal at 1073 cm${}^{-1}$ on 12 random locations of the template. Thus, we calculated the relative standard deviation (RSD; see error bars in Figure 4c), and we reached a uniform SERS signal with an average RSD inferior to 18% for all experimental data. We also calculated the enhancement factor (EF) for tilted and vertical nanocolumns at LOD with this expression [63]:

(1) $$\begin{array}{c} EF=\frac{I_{SERS}}{I_{Raman}}\times \frac{C_{Raman}}{C_{SERS}} \end{array}$$

where $C_{SERS}$ (10 or 20 nM) and $C_{Raman}$ (1 M) are, respectively, the concentrations of thiophenol at LOD for gold nanocolumns and glass substrate without any gold nanocolumns. $I_{SERS}$ and $I_{Raman}$ are, respectively, the intensities of the SERS and Raman signals at each excitation wavelength (see the SERS spectra in Figure 4a,b and Raman spectra displayed in Figure S1 in Supplementary Materials). We achieved EF values of 4.8 × 10${}^{8}$ for tilted nanocolumns and 9.8 × 10${}^{8}$ for vertical nanocolumns, which are larger than those recorded with other plasmonic nanosystems for thiophenol detection in the scientific literature (Table 2).

### 3.3. Adsorption of Thiophenol on the Gold Vertical and Tilted Nanocolumnar Templates

To determine the adsorption properties of Au vertical and tilted nanocolumns, we treated the experimental data with the following Langmuir model [48,49,50]:

(2) $$\begin{array}{c} I=I_{max}\frac{K_{ads}C_{Th}}{1+K_{ads}C_{Th}} \end{array}$$

where *I* is the intensity of the SERS signal at the thiophenol concentration C${}_{Th}$. $I_{max}$ is the maximum intensity of the SERS signal corresponding to the deposition of a thiophenol monolayer. Lastly, $K_{ads}$ is the adsorption constant. We found good agreements between experimental data and Langmuir fits (see the red and blue Langmuir fits in Figure 4c). From these Langmuir fits, we extracted the values of $K_{ads}$ (see Table 3). These latter are higher than those observed with commercial templates composed of gold-inverted pyramid structures, which are benchmark SERS templates called Klarite substrates (see Table 3) [49]. Moreover, from previously determined values of $K_{ads}$, we also calculated the free energy of adsorption, $\Delta G_{ads}$, by employing this formula [48,66]:

(3) $$\begin{array}{c} \Delta G_{ads}=-RT\times ln\left(K_{ads}\right) \end{array}$$

where *R* and *T* represent the ideal gas constant and the temperature, respectively. We observed a higher free energy of adsorption for our SERS templates compared to the Klarite substrates for thiophenol detection (see Table 3). These values of $K_{ads}$ and $\Delta G_{ads}$ denoted a higher adsorption of thiophenol (benzenethiol) towards Au nanocolumnar templates than for the commercial Klarite substrates. Furthermore, these values of $K_{ads}$ and $\Delta G_{ads}$ also proved that the benzenethiolate–gold system was an equilibrium system.

To complete this investigation, we displayed the intensity of the SERS signal at 1073 cm${}^{-1}$ versus the number of detected molecules (see Figure 4d). We calculated the number of detected molecules $N_{det}$ (here, thiophenol) with the following formula:

(4) $$\begin{array}{c} N_{det}=N_{Avo}\times \sigma_{surface}\times S_{illu} \end{array}$$

where $N_{Avo}$ depicts the Avogadro number equal to 6.022 × 10${}^{23}$ mol${}^{-1}$. $S_{illu}$ here describes the surface illuminated by the laser spot whose diameter is around 720 nm at $\lambda_{exc}$ of 532 nm, and 860 nm at $\lambda_{exc}$ of 633 nm [67] ($S_{illu}$ = 4.1 × 10${}^{5}$ nm${}^{2}$ and 5.8 × 10${}^{5}$ nm${}^{2}$ at $\lambda_{exc}$ of 532 nm and 633 nm, respectively). Lastly, $\sigma_{surface}$ corresponds to the surface coverage of thiophenol calculated with this formula [48]:

(5) $$\begin{array}{c} \sigma_{surface}=\sigma_{max}\frac{K_{ads}C_{Th}}{1+K_{ads}C_{Th}} \end{array}$$

where $\sigma_{max}$ represents the surface coverage for a monolayer of benzenethiol ($\sigma_{max}$≈ 5.44 × 10${}^{-10}$ mol/cm${}^{2}$ [68]). $K_{ads}$ and $C_{Th}$ have been defined previously, and each $K_{ads}$ value was calculated from Figure 4c and is shown in Table 3. From Figure 4d, we observed a linear variation of the SERS intensity when $N_{det}$ increases for each gold nanocolumnar template. Moreover, the slope (related to the sensitivity) is higher for the gold vertical nanocolumnar template. We associate this behavior with the fact that the plasmonic resonance present in the vertical nanocolumns is more intense and better defined, as discussed above (Section 3.1), which therefore gives rise to better localized and more intense electric field hotspots in the nanocolumn gaps.

## 4. Conclusions

Herein, we have evidenced an excellent SERS detection of thiophenol molecules using vertical and tilted gold nanocolumns fabricated with a low-cost technique, namely GLAD with magnetron sputtering. We focused on thiophenol analyte due to its strong adsorption affinity on metals. We achieved values of the adsorption constant $K_{ads}$ from 1.8 × 10${}^{6}$ M${}^{-1}$ to 2.0 × 10${}^{6}$ M${}^{-1}$ as well as values of adsorption free energy $\Delta G_{ads}$ from −35.9 kJ/mol to −36.2 kJ/mol for thiophenol on our gold nanocolumnar templates. Moreover, enhancement factors of 4.8 × 10${}^{8}$ and 9.8 × 10${}^{8}$ were obtained for tilted and vertical gold nanocolumns, respectively. All of these values are greater than those exhibited by the different SERS templates in the scientific literature cited in this work. Detection threshold limits of 10 nM and 20 nM were attained for vertical and tilted gold nanocolumns, respectively. Furthermore, we evidenced a uniform SERS signal regarding gold nanocolumnar templates with an average RSD < 18%. Henceforward, this work can open the way to a commercial production of these SERS templates, as happens nowadays with Klarite ones, with a low-cost and quick method of manufacturing on large surfaces.

## Figures and Tables

**Table 1:** Raman peaks of the thiophenol molecule.

Raman Shift (cm^−1^)	Vibration Modes
999	C–H out-of-plane bending, ring out-of-plane deformation
1022	Ring in-plane deformation, C–C symmetric stretching
1073	C–C symmetric stretching, C–S stretching

**Table 2:** Values of the enhancement factor (EF) obtained with our SERS templates (NCs = nanocolumns; AuNPs = gold nanoparticles) compared to other plasmonic substrates for thiophenol detection.

SERS Template	Enhancement Factor (EF)	References
Vertical Au NCs	9.8 × 10${}^{8}$	This work
Tilted Au NCs	4.8 × 10${}^{8}$	This work
Au nanostar array on silver film	1.9 × 10${}^{8}$	[64]
Au nanogap arrays	1.5 × 10${}^{6}$	[46]
Au nanogap between AuNPs and Au layer	8.5 × 10${}^{2}$	[47]
AuNPs on single-layer porous silicon film	∼10${}^{2}$	[65]

**Table 3:** Values of $K_{ads}$ and $\Delta G_{ads}$ obtained with our SERS templates (NCs = Nanocolumns) compared to commercial Klarite substrates, at the same temperature of 300 K.

SERS Template	Adsorption Constant ($K_{\mathrm{ads}}$)	Free Energy of Adsorption ($\Delta G_{\mathrm{ads}}$)
Vertical Au NCs	2.0 × 10${}^{6}$ M${}^{-1}$	−36.2 kJ/mol
Tilted Au NCs	1.8 × 10${}^{6}$ M${}^{-1}$	−35.9 kJ/mol
Klarite	1.1 × 10${}^{6}$ M${}^{-1}$	−34.7 kJ/mol

## Data Availability

The data presented in this study are available on reasonable request to the author.

## Supplementary Materials

The following are available online at https://www.mdpi.com/article/10.3390/nano12234157/s1, Figure S1: Raman spectra of thiophenol molecules at the concentration of 1 M recorded on a glass substrate without any gold nanocolumns at excitation wavelengths of 532 nm and 633 nm.

## References

[1] Moskovits M. (1985). Surface-enhanced spectroscopy. Rev. Mod. Phys..

[2] Nie S., Emory S.R. (1997). Probing Single Molecules and Single Nanoparticles by Surface-Enhanced Raman Scattering. Science.

[3] Xu H., Bjerneld E.J., Käll M., Börjesson L. (1999). Spectroscopy of Single Hemoglobin Molecules by Surface Enhanced Raman Scattering. Phys. Rev. Lett..

[4] Lim D.-K., Jeon K.-S., Kim H.M., Nam J.-M., Suh Y.D. (2010). Nanogap-engineerable Raman-active nanodumbbells for single-molecule detection. Nat. Mater..

[5] Nam J.-M., Oh J.-W., Lee H., Suh Y.D. (2016). Plasmonic Nanogap-Enhanced Raman Scattering with Nanoparticles. Acc. Chem. Res..

[6] Yue W., Wang Z., Whittaker J., Lopez-Royo F., Yang Y., Zayats A.V. (2017). Amplification of surface-enhanced Raman scattering due to substrate-mediated localized surface plasmons in gold nanodimers. J. Mater. Chem. C.

[7] Graniel O., Iatsunskyi I., Coy E., Humbert C., Barbillon G., Michel T., Maurin D., Balme S., Miele P., Bechelany M. (2019). Au-covered hollow urchin-like ZnO nanostructures for surface-enhanced Raman scattering sensing. J. Mater. Chem. C.

[8] Banholzer M.J., Millstone J.E., Qin L., Mirkin C.A. (2008). Rationally designed nanostructures for surface-enhanced Raman spectroscopy. Chem. Soc. Rev..

[9] Lal S., Grady N.K., Kundu J., Levin C.S., Lassiter J.B., Halas N.J. (2008). Tailoring plasmonic substrates for surface enhanced spectroscopies. Chem. Soc. Rev..

[10] Sonntag M.D., Klingsporn J.M., Zrimsek A.B., Sharma B., Ruvuna L.K., Van Duyne R.P. (2014). Molecular plasmonics for nanoscale spectroscopy. Chem. Soc. Rev..

[11] Ding S.-Y., You E.-M., Tian Z.-Q., Moskovits M. (2017). Electromagnetic theories of surface-enhanced Raman spectroscopy. Chem. Soc. Rev..

[12] Barbillon G. (2022). Latest Advances in Metasurfaces for SERS and SEIRA Sensors as Well as Photocatalysis. Int. J. Mol. Sci..

[13] Quilis N.G., Hageneder S., Fossati S., Auer S.K., Venugopalan P., Bozdogan A., Petri C., Moreno-Cencerrado A., Toca-Herrera J.L., Jonas U. (2020). UV-Laser Interference Lithography for Local Functionalization of Plasmonic Nanostructures with Responsive Hydrogel. J. Phys. Chem. C.

[14] Dhawan A., Duval A., Nakkach M., Barbillon G., Moreau J., Canva M., Vo-Dinh T. (2011). Deep UV nano-microstructuring of substrates for surface plasmon resonance imaging. Nanotechnology.

[15] Barbillon G., Faure A.-C., El Kork N., Moretti P., Roux S., Tillement O., Ou M.G., Descamps A., Perriat P., Vial A. (2008). How nanoparticles encapsulating fluorophores allow a double detection of biomolecules by localized surface plasmon resonance and luminescence. Nanotechnology.

[16] Barbillon G., Bijeon J.-L., Bouillard J.-S., Plain J., Lamy de La Chapelle M., Adam P.-M., Royer P. (2008). Detection in near-field domain of biomolecules adsorbed on a single metallic nanoparticle. J. Microsc..

[17] Manfrinato V.R., Camino F.E., Stein A., Zhang L.H., Lu M., Stach E.A., Black C.T. (2019). Patterning Si at the 1 nm Length Scale with Aberration-Corrected Electron-Beam Lithography: Tuning of Plasmonic Properties by Design. Adv. Funct. Mater..

[18] Farcau C., Marconi D., Colnita A., Brezestean I., Barbu-Tudoran L. (2019). Gold Nanospot-Shell Arrays Fabricated by Nanoimprint Lithography as a Flexible Plasmonic Sensing Platform. Nanomaterials.

[19] Chau Y.F.C., Chen K.H., Chiang H.P., Lim C.M., Huang H.J., Lai C.H., Kumara N.T.R.N. (2019). Fabrication and Characterization of a Metallic-Dielectric Nanorod Array by Nanosphere Lithography for Plasmonic Sensing Applications. Nanomaterials.

[20] Barbillon G., Noblet T., Busson B., Tadjeddine A., Humbert C. (2018). Localised detection of thiophenol with gold nanotriangles highly structured as honeycombs by nonlinear sum frequency generation spectroscopy. J. Mater. Sci..

[21] Lao Z., Zheng Y., Dai Y., Hu Y., Ni J., Ji S., Cai Z., Smith Z.J., Li J., Zhang L. (2020). Nanogap Plasmonic Structures Fabricated by Switchable Capirally-Force Driven Self-Assembly for Localized Sensing of Anticancer Medicines with Microfluidic SERS. Adv. Funct. Mater..

[22] Yin Z., Zhou Y., Cui P., Liao J., Rafailovich M.H., Sun W. (2020). Fabrication of ordered bi-metallic array with superstructure of gold micro-rings via templated-self-assembly procedure and its SERS applications. Chem. Commun..

[23] Fusco Z., Bo R., Wang Y., Motta N., Chen H., Tricoli A. (2019). Self-assembly of Au nano-islands with tuneable organized disorder for highly sensitive SERS. J. Mater. Chem. C.

[24] Wu P., Zhong L.-B., Liu Q., Zhou X., Zheng Y.-M. (2019). Polymer induced one-step interfacial self-assembly method for the fabrication of flexible, robust and free-standing SERS substrates for rapid on-site detection of pesticide residues. Nanoscale.

[25] Mark A.G., Gibbs J.G., Lee T.-C., Fischer P. (2013). Hybrid nanocolloids with programmed three-dimensional shape and material composition. Nat. Mater..

[26] Huang Z., Bai F. (2014). Wafer-scale, three-dimensional helical porous thin films deposited at a glancing angle. Nanoscale.

[27] Liu Y., Liu J., Sohn S., Li Y., Cha J.J., Schroers J. (2015). Metallic glass nanostructures of tunable shape and composition. Nat. Commun..

[28] Ai B., Zhao Y. (2019). Glancing angle deposition meets colloidal lithography: A new evolution in the design of nanostructures. Nanophotonics.

[29] Han J.-H., Kim D., Kim J., Kim G., Fischer P., Jeong H.-H. (2022). Plasmonic Nanostructure Engineering with Shadow Growth. Adv. Mater..

[30] Eiamchai P., Chananonnawathorn C., Horprathum M., Patthanasettakul V., Limwichean S., Nuntawong N. (2020). Spatial elemental investigations in nanostructured alloyed Ag/Au SERS substrates by magnetron sputtering oblique-angle co-deposition towards increased performance and shelf life. Appl. Surf. Sci..

[31] Kelly P.J., Arnell R.D. (2000). Magnetron sputtering: A review of recent developments and applications. Vacuum.

[32] Robbie K., Brett M.J., Lakhtakia A. (1996). Chiral sculptured thin films. Nature.

[33] Abelmann L., Lodder C. (1997). Oblique evaporation and surface diffusion. Thin Solid Films.

[34] He Y., Zhao Y. (2011). Advanced multi-component nanostructures designed by dynamic shadowing growth. Nanoscale.

[35] Barranco A., Borras A., Gonzalez-Elipe A.R., Palmero A. (2016). Perspectives on oblique angle deposition of thin films: From fundamentals to devices. Prog. Mater. Sci..

[36] Díaz-Nuñez P., García-Martín J.M., González M.U., González-Arrabal R., Rivera A., Alonso-González P., Martín-Sánchez J., Taboada-Gutiérrez J., González-Rubio G., Guerrero-Martínez A. (2019). On the Large Near-Field Enhancement on Nanocolumnar Gold Substrates. Sci. Rep..

[37] Gahlaut S.K., Savargaonkar D., Sharan C., Yadav S., Mishra P., Singh J.P. (2020). SERS Platform for Dengue Diagnosis from Clinical Samples Employing a Hand Held Raman Spectrometer. Anal. Chem..

[38] Kang Y., Xue X., Wang W., Fan Y., Li W., Ma T., Zhao F., Zhang Z. (2020). Design of Armrest Ag Nanorod Arrays with High SERS Performance for Sensitive Biomolecule Detection. J. Phys. Chem. C.

[39] Lawson Z.R., Preston A.S., Korsa M.T., Dominique N.L., Tuff W.J., Sutter E., Camden J.P., Adam J., Hughes R.A., Neretina S. (2022). Plasmonic Gold Trimers and Dimers with Air-Filled Nanogaps. ACS Appl. Mater. Interfaces.

[40] Yang Y., Hu Z., Wang Y., Wang B., Zhan Q., Zhang Y., Ao X. (2016). Broadband SERS substrates by oblique angle deposition method. Opt. Mater. Express.

[41] Chu H.O., Song S., Li C., Gibson D. (2017). Surface Enhanced Raman Scattering Substrates Made by Oblique Angle Deposition: Methods and Applications. Coatings.

[42] Sha P., Su Q., Dong P., Wang T., Zhu C., Gao W., Wu X. (2021). Fabrication of Ag@Au (core@shell) nanorods as a SERS substrate by the oblique angle deposition process and sputtering technology. RSC Adv..

[43] Tsen C.-M., Yu C.-W., Chen S.-Y., Lin C.-L., Chuang C.-Y. (2021). Application of surface-enhanced Raman scattering in rapid detection of dithiocarbamate pesticide residues in foods. Appl. Surf. Sci..

[44] Yadav S., Khanam R., Singh J.P. (2022). A purview into highly sensitive magnetic SERS detection of hemozoin biomarker for rapid malaria diagnosis. Sens. Actuator B Chem..

[45] Barbillon G., Noblet T., Humbert C. (2020). Highly crystalline ZnO film decorated with gold nanospheres for PIERS chemical sensing. Phys. Chem. Chem. Phys..

[46] Le-The H., Lozeman J.J.A., Lafuente M., Munoz P., Bomer J.G., Duy-Tong H., Berenschot E., van den Berg A., Tas N.R., Odijk M. (2019). Wafer-scale fabrication of high-quality tunable gold nanogap arrays for surface-enhanced Raman scattering. Nanoscale.

[47] Ding T., Herrmann L.O., de Nijs B., Benz F., Baumberg J.J. (2015). Self-Aligned Colloidal Lithography for Controllable and Tuneable Plasmonic Nanogaps. Small.

[48] Tripathi A., Emmons E.D., Fountain A.W., Guicheteau J.A., Moskovits M., Christesen S.D. (2015). Critical Role of Adsorption Equilibria on the Determination of Surface-Enhanced Raman Enhancement. ACS Nano.

[49] Tripathi A., Emmons E.D., Kline N.D., Christesen S.D., Fountain A.W., Guicheteau J.A. (2018). Molecular Structure and Solvent Factors Influencing SERS on Planar Gold Substrates. J. Phys. Chem. C.

[50] Emmons E.D., Guicheteau J.A., Fountain A.W., Tripathi A. (2020). Effect of substituents on surface equilibria of thiophenols and isoquinolines on gold substrates studied using surface-enhanced Raman spectroscopy. Phys. Chem. Chem. Phys..

[51] Barbillon G., Ivanov A., Sarychev A.K. (2021). SERS Amplification in Au/Si Asymmetric Dimer Array Coupled to Efficient Adsorption of Thiophenol Molecules. Nanomaterials.

[52] Barbillon G., Graniel O., Bechelany M. (2021). Assembled Au/ZnO Nano-Urchins for SERS Sensing of the Pesticide Thiram. Nanomaterials.

[53] Sit J.C., Vick D., Robbie K., Brett M.J. (1999). Thin Film Microstructure Control Using Glancing Angle Deposition by Sputtering. J. Mater. Res..

[54] Garcia-Martin J.M., Alvarez R., Romero-Gomez P., Cebollada A., Palmero A. (2010). Tilt angle control of nanocolumns grown by glancing angle sputtering at variable argon pressures. Appl. Phys. Lett..

[55] Alvarez R., Garcia-Martin J.M., Lopez-Santos M.C., Rico V., Ferrer F.J., Cotrino J., Gonzalez-Elipe A.R., Palmero A. (2014). On the Deposition Rates of Magnetron Sputtered Thin Films at Oblique Angles. Plasma Process. Polym..

[56] Alvarez R., Garcia-Martin J.M., Macias-Montero M., Gonzalez-Garcia L., Gonzalez J.C., Rico V., Perlich J., Cotrino J., Gonzalez-Elipe A.R., Palmero A. (2013). Growth regimes of porous gold thin films deposited by magnetron sputtering at oblique incidence: From compact to columnar microstructures. Nanotechnology.

[57] Alvarez R., Garcia-Valenzuela A., Rico V., Garcia-Martin J.M., Cotrino J., Gonzalez-Elipe A.R., Palmero A. (2019). Kinetic energy-induced growth regimes of nanocolumnar Ti thin films deposited by evaporation and magnetron sputtering. Nanotechnology.

[58] Vitrey A., Alvarez R., Palmero A., Gonzalez M.U., Garcia-Martin J.M. (2017). Fabrication of black-gold coatings by glancing angle deposition with sputtering. Beilstein J. Nanotechnol..

[59] Christopher Love J., Estroff L.A., Kriebel J.K., Nuzzo R.G., Whitesides G.M. (2005). Self-Assembled Monolayers of Thiolates on Metals as a Form of Nanotechnology. Chem. Rev..

[60] Barbillon G. (2022). Au Nanoparticles Coated ZnO Film for Chemical Sensing by PIERS Coupled to SERS. Photonics.

[61] Li S., Wu D., Xu X., Gu R. (2007). Theoretical and experimental studies on the adsorption behavior of thiophenol on gold nanoparticles. J. Raman Spectrosc..

[62] Tetsassi Feugmo C.G., Liégeois V. (2013). Analyzing the vibrational signatures of thiophenol adsorbed on small gold clusters by DFT calculations. ChemPhysChem.

[63] Le Ru E.C., Blackie E.J., Meyer M., Etchegoin P.G. (2007). Surface enhanced Raman scattering enhancement factors: A comprehensive study. J. Phys. Chem. C.

[64] Park S., Lee J., Ko H. (2017). Transparent and Flexible Surface-Enhanced Raman Scattering (SERS) Sensors Based on Gold Nanostar Arrays Embedded in Silicon Rubber Film. ACS Appl. Mater. Interfaces.

[65] Jiao Y., Koktysh D.S., Phambu N., Weiss S.M. (2010). Dual-mode sensing platform based on colloidal gold functionalized porous silicon. Appl. Phys. Lett..

[66] Karpovich D.S., Blanchard G.J. (1994). Direct Measurement of the Adsorption Kinetics of Alkanethiolate Self-Assembled Monolayers on a Microcrystalline Gold Surface. Langmuir.

[67] Alvarez-Puebla R.A. (2012). Effects of the Excitation Wavelength on the SERS Spectrum. J. Phys. Chem. Lett..

[68] Caldwell J.D., Glembocki O., Bezares F.J., Bassim N.D., Rendell R.W., Feygelson M., Ukaegbu M., Kasica R., Shirey L., Hosten C. (2011). Plasmonic Nanopillar Arrays for Large-Area, High-Enhancement Surface-Enhanced Raman Scattering Sensors. ACS Nano.

