# Supplementary material for: Gold Nanocolumnar Templates for Effective Chemical Sensing by Surface-Enhanced Raman Scattering
Source: Nanomaterials (Basel). 2022 Nov 24;12(23):4157. doi: 10.3390/nano12234157 (PMC9741134; doi:10.3390/nano12234157)
Supplement: Supplementary file 1 [file nanomaterials-12-04157-s001.zip › nanomaterials-2028724-supplementary.pdf]

Supplementary Materials

Gold Nanocolumnar Templates for Effective Chemical Sensing by Surface-Enhanced Raman Scattering

Grégory Barbillon[1,*], Christophe Humbert[2], María Ujué González[3], José Miguel García-Martín[3]

[1] EPF-École d'Ingénieurs, 55 Avenue du Président Wilson, 94230 Cachan, France.
[2] Institut de Chimie Physique, Université Paris-Saclay, CNRS, UMR8000, 91405 Orsay, France.
[3] Instituto de Micro y Nanotecnología, IMN-CNM, CSIC (CEI UAM+CSIC), Isaac Newton 8, 28760, Tres Cantos, Madrid, Spain.
*Correspondence : gregory.barbillon@epf.fr

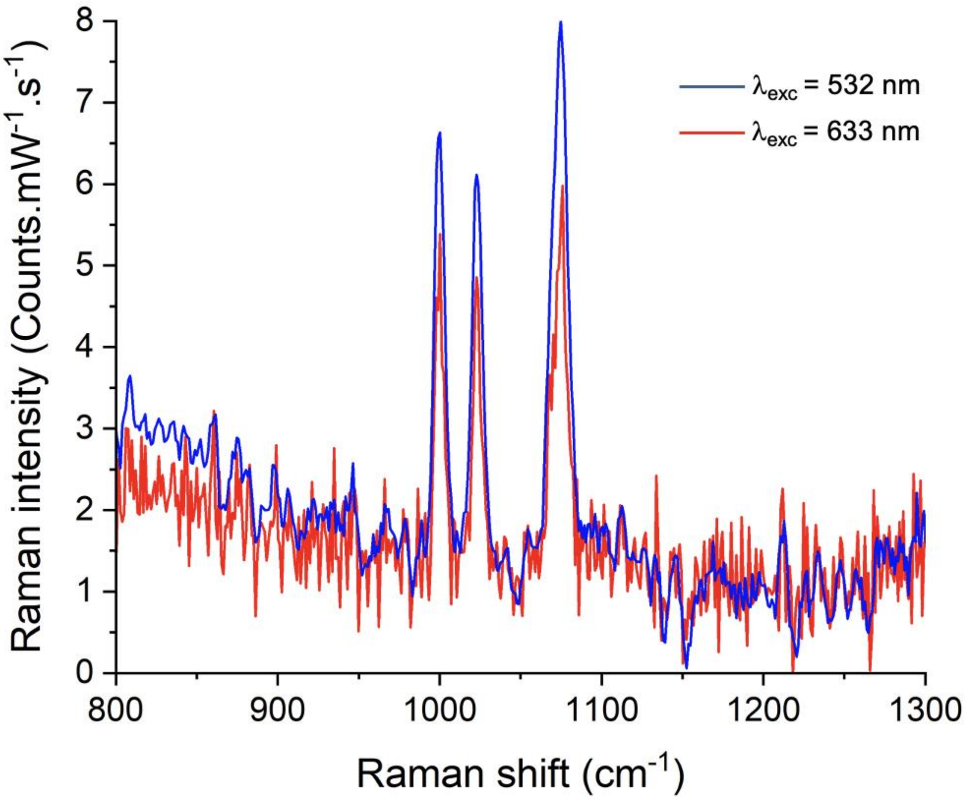

Figure S1. Raman spectra of thiophenol molecules at the concentration of 1 M recorded on a glass substrate without any gold nanocolumns at excitation wavelengths of 532 nm (in blue color) and 633 nm (in red color).
